# Resistin Stimulates Expression of Chemokine Genes in Chondrocytes via Combinatorial Regulation of C/EBPβ and NF-κB

**DOI:** 10.3390/ijms151017242

**Published:** 2014-09-26

**Authors:** Ziji Zhang, Zhiqi Zhang, Yan Kang, Changhe Hou, Xin Duan, Puyi Sheng, Linda J. Sandell, Weiming Liao

**Affiliations:** 1 Department of Orthopaedic Surgery, the First Affiliated Hospital of Sun Yat-sen University, Guangzhou 510080, China; E-Mails: zhangziji1984@163.com (Zi.Z.); zhzhiqi@163.com (Zh.Z.); neokang@163.com (Y.K.); changhe713@sina.com (C.H.); xind@wudosis.wustl.edu (X.D.); shengpuyi@hotmail.com (P.S.); 2 Department of Orthopaedic Surgery, Washington University School of Medicine at Barnes-Jewish Hospital, St. Louis, MO 63110, USA

**Keywords:** resistin, chondrocytes, chemokines, transcription factors

## Abstract

To further investigate the regulation role of two chemokine genes *CCL3* and *CCL4* in chondrocytes in response to *resistin*, human primary chondrocytes and *T/C-28a2* cells were cultured. The function of *resistin* on the chemokine genes, and the expression of *C/EBPβ*, *NF-κB* isoforms were tested using qPCR. The methods used to investigate timed co-regulation of *C/EBPβ* and *NF-κB*were *NF-κB* inhibitor (*IKK-NBD*) and *C/EBPβ* inhibitor (*SB303580*) treatments, and subcellular localization, with or without *resistin* stimulation. Results showed that *resistin* could increase the up-regulation of chemokine genes independently. *Resistin* increased the expression of *C/EBPβ* and *NF-κB* isoforms. *C/EBPβ* regulated basal activity and steadily increased over time up to 24h with *resistin*. *NF-κB* was up-regulated upon induction with *resistin*, peaking at 4 h. *C/EBPβ* and *NF-κB* co-enhanced the chemokines expression; inhibition of their activity was additive. The timing of activation in chondrocytes was confirmed by subcellular localization of *C/EBPβ* and *c-rel*. Chondrocytes react to *resistin* in a non-restricted cell-specific manner, utilizing *C/EBPβ* and *NF-κB* in a combinatorial regulation of chemokine gene expression. The activity of *C/EBPβ* is augmented by a transient increase in activity of *NF-κB*, and both transcription factors act independently on the chemokine genes, *CCL3* and *CCL4*. Thus, *resistin* stimulates *CCL3* and *CCL4* through combinatorial regulation of *C/EBPβ* and *NF-κB* in chondrocytes.

## 1. Introduction

*Resistin*, an adipokine, is expressed in rheumatoid arthritis (RA) or osteoarthritis (OA) patients and in synovial joints after injury [1,2,3,4], and the plasma *resistin* level is significantly correlated with erythrocyte sedimentation rate (ESR) and C reactive protein (CRP) [3]. As a 12.5 kDa cysteine-rich polypeptide, *resistin* is expressed not only by human adipocytes, but also in high levels by macrophages [5,6]. *Resistin* can up-regulate interleukin *(IL)-1*, *IL-6* and tumor necrosis factor α (*TNF-α*) and is present in blood samples and the synovial fluid of patients with RA. Intra-articular injection of *resistin* induces arthritis in healthy mouse joints [7]. Although many aspects of the biological effects and the regulation of *resistin* remain controversial, our previous study indicated that *resistin* stimulates a large set of chemokines in chondrocytes that are known to be important in inflammatory diseases, including RA and OA [8,9,10,11].

Chemokines are a specific class of cytokines that typically mediate chemoattraction (chemotaxis) between cells. There are two major subclasses having conserved cysteine residues either adjacent (CC) or separated by one amino acid (CXC) [12]. In adult normal cartilage and OA patients, we have shown that a large set of chemokine genes is up-regulated by the pro-inflammatory cytokine *IL-1β* and *resistin* [8,13]. It can be expected that this increase in a wide range of chemokines will have a significant impact on cartilage cells and other related joint tissues; it should be considered in the pathophysiology of OA.

As we reported, *resistin* increased expression of *IL-1β*, *CCL3*, *CCL3L1*, *CCL4*, *CCL5*, *CCL8*, *CCL20*, *CXCL1*, *CXCL2*, *CXCL3*, *CXCL5*, *CXCL6*, and *CXCL8* (*IL-8*) over 10-fold in human articular chondrocytes [8]. A computational analysis of these co-regulated genes identified *NF-κB*, *C/EBPβ* and myocyte enhancer binding factor 3 (*MEF-3*), as candidate transcriptional regulators. We have shown that *C/EBPβ* and *NF-κB* are associated with IL-1β-induced up-regulation of chemokine genes in chondrocytes [14]. Furthermore, we demonstrated that *C/EBPβ*, *NF-κB* and mRNA stability are involved the up-regulation of chemokines by human chondrocytes in response to *resistin* and that *resistin* increases chemokine genes expression at both the transcriptional and post-transcriptional levels in temporal patterns similar to the *IL-1β* patterns [13].

In the present study, we further investigated the mechanism of the co-regulatory roles of transcription (*C/EBPβ* and *NF-κB*) in the up-regulation of two chemokine genes *CCL3* (macrophage inflammatory protein 1α, *MIP-1α*) and *CCL4* (*MIP-1β*) in chondrocytes in response to *resistin*.

## 2. Results and Discussion

### 2.1. Resistin Independently Stimulates the Expression of Chemokine Genes in Normal Chondrocytes

**Figure 1:**
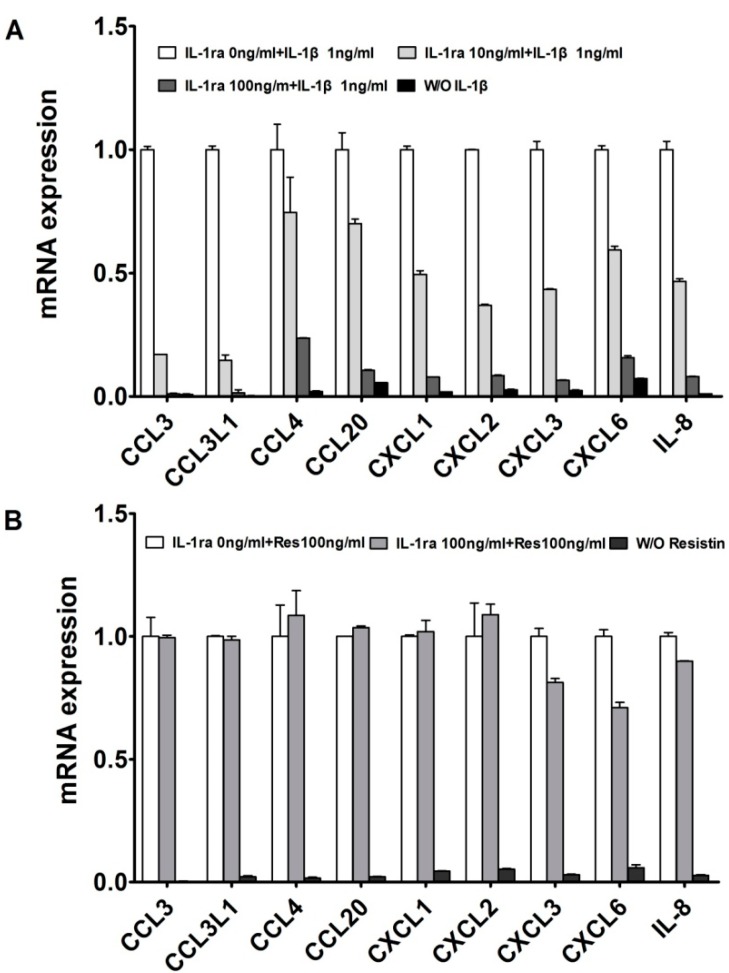
*Resistin* independently stimulates the expression of chemokines in normal human articular chondrocytes (HACs). (**A**) HACs were treated with 1 ng/mL *IL-1β* with various doses of *IL-1ra* for 24 h, as indicated. W/O *IL-1β* means blank control without IL-1β; (**B**) HACs were treated with 100 ng/mL *resistin* with or without *IL-1ra* for 24 h, as indicated. W/O *resistin* means blank control without *resistin*. The relative expression levels were examined by quantitative real time PCR method. Each bar represents the mean ± S.D. from three experiments. W/O *resistin* means blank control without *resistin*.

We reported that in adult normal cartilage and OA patients, a large set of chemokines are up-regulated by the *resistin* or *IL-1β*; and *resistin* can stimulated the expression of *IL-1β* [8,13,14]. Figure 1 illustrates our treatments with effective *IL-1* receptor antagonist Recombinant Human *IL-1ra*. The lower concentration (10 ng/mL) of *IL-1ra* only partially inhibited the IL-1-induced responses, whereas the more effective concentration (100 ng/mL) almost suppressed all tested cytokine and chemokine genes. Meanwhile, the higher concentration (100 ng/mL) of *IL-1ra* was tested against *resistin* in panel 1B. In this experiment, cytokine and chemokine genes were still significantly increased in human articular chondrocytes in response to *resistin* (Figure 1B). This further suggests that the effects of *resistin*are not dependent on stimulation of *IL-1*.

### 2.2. Resistin Stimulates the Expression of C/EBPβ in Chondrocytes

**Figure 2:**
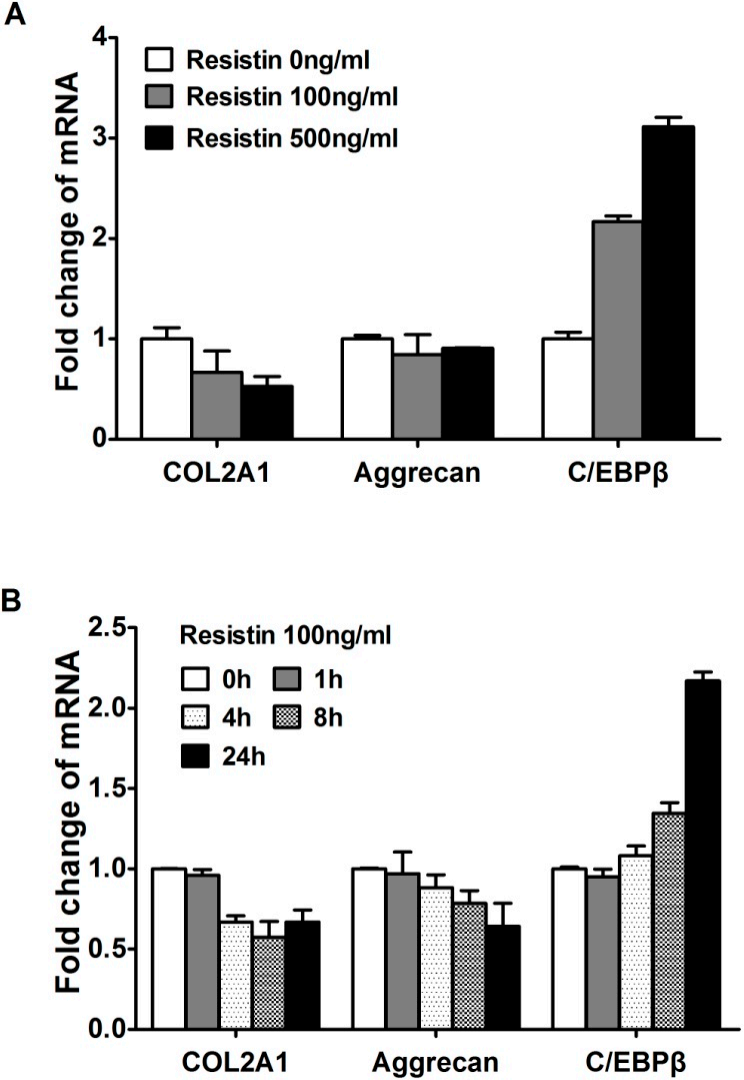
*Resistin* stimulates the expression of *C/EBPβ* in normal human articular chondrocytes (HACs). The relative expression levels were examined in normal chondrocytes from human articular cartilage treated with *resistin* for various doses and times using quantitative real time PCR method. (**A**) Cells were treated with various concentrations of *resistin* for 24 h as indicated in HACs; (**B**) Cells were treated with 100 ng/mL *resistin* for various times as indicated in HACs. Each bar represents the mean ± S.D. from three experiments.

Computational analysis and co-transfection of *C/EBPβ* previously suggested that *C/EBPβ* was involved in the *resistin*-induced up-regulation of chemokine genes [14]. The down-regulation of *COL2A1* and *aggrecan* genes and up-regulation of *C/EBPβ* showed a dose response (Figure 2A). There is no statistic difference (*p* > 0.05) referring to the changes of *COL2A1* and *aggrecan*, but with opposite trend to *CEBPβ*. When cells were stimulated with 100 ng/mL *resistin* over 24 h, the expression level of *C/EBPβ* mRNA was gradually increased to about 3-fold (Figure 2B). *C/EBPβ* repressed expression *COL2A1* mRNA to about 60% within 24 h in human chondrocytes as expected (Figure 2B) as we have shown previously for rat chondrosarcoma cells [15] and human articular chondrocytes [8,13,14]. We referred the results of *COL2A1* and *ACAN* to illuminate that they can be restrained by *C/EBPβ*, and its tendency is opposite to *C/EBPβ*, providing another aspect to prove the function of *resistin* on *C/EBPβ*.

### 2.3. NF-κB Is Involved in the Up-Regulation of Chemokine Genes by Chondrocytes in Response to Resistin

**Figure 3:**
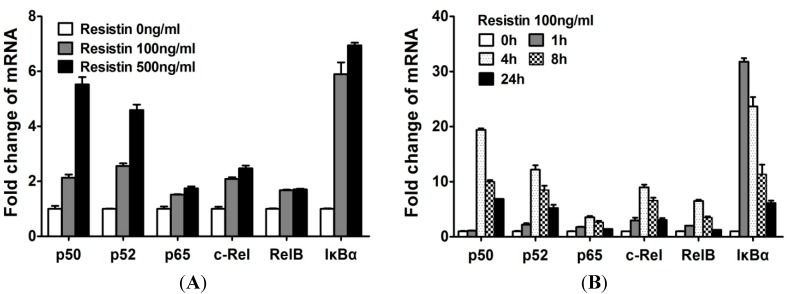
*Resistin* stimulates the expression of *NF-κB* isoforms in normal human articular chondrocytes (HACs). The relative expression levels were examined in normal chondrocytes from human articular cartilage treated with *resistin* for various doses and times using quantitative real time PCR method. (**A**) Cells were treated with various concentrations of *resistin* for 24 h as indicated in HACs; (**B**) Cells were treated with 100 ng/mL *resistin* for various times as indicated in HACs. Each bar represents the mean ± S.D. from three experiments.

As the transcription complex *NF-κB* has been shown to be involved in the *resistin*-induced up-regulation of multiple cytokine and chemokine genes by the co-transfection of *NF-κB* and *C/EBPβ*, and by *IKK-NBD* inhibition, we sought to determine the relative roles of *C/EBPβ* and *NF-κB*. The expression of *NF-κB* in normal human articular chondrocytes was examined. The levels of *NF-κB1* (*p50*), *NF-κB2* (*p52*), *RelA* (*p65*), *c-Rel*, *RelB*, *IκBα* mRNAs were investigated in resistin-treated chondrocytes at various doses and times. The levels of mRNA encoding *NF-κB* isoforms increased in a dose-dependent manner in response to *resistin* (Figure 3A). *NF-κB* isoforms were rapidly increased, but they decreased after 4 h (Figure 3B), which was the same kinetics as we previously reported for *NF-κB* function by using *pNF-κB* luciferase reporter over 24 h in response to *resistin* [8]. When compared to the concentration of *C/EBPβ* in cells, we show that the *NF-κB* isoforms are lower. In human chondrocytes, ∆*C* of *C/EBPβ* is average 1.73 and thus lower than ∆*C* of *NF-κB1* (5.33), *NF-κB2* (5.46), *RelA* (18.23), *c-Rel* (6.89) and *RelB* (7.04). However, these studies also provided insight into the likely *NF-κB* subunits used in chondrocytes in response to *resistin*. *p50*, *p52* and *c-Rel* showed the highest relative increase. Therefore, these results indicated that *NF-κB* is involved in the expression of chemokines, particularly at 4 to 8 h of exposure.

### 2.4. C/EBPβ and NF-κB Co-Enhance the Expression of CCL3 and CCL4 in Human Chondrocytes in Response to Resistin

**Figure 4:**
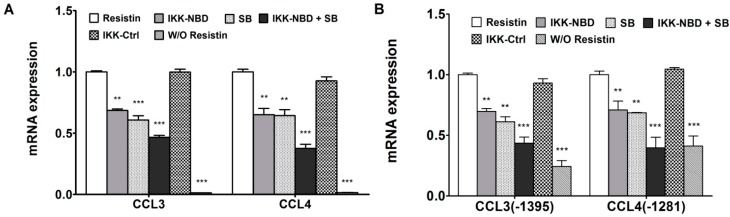
*IKK-NBD* and *SB303580* (*SB*) co-enhance the inhibition of chemokines by human chondrocytes with *resistin* treatment. (**A**) Human articular chondrocytes (HAC) were pretreated with vehicle (*DMSO*), *IKK-NBD* peptide (100 μM), *SB303580* (100 μM), or *IKK-NBD* control peptide (100 μM) for 1 h and then exposed to *resistin* (100 ng/mL) for 4 h. After *resistin* treatment, total *RNA* was isolated, and real-time quantitative-PCR was performed; (**B**) The *CCL3* (−1395) and *CCL4* (−1281) promoter constructs were transfected into *T/C-28a2* cells and incubated for 24 h, then were pretreated with vehicle (*DMSO*), *IKK-NBD* peptide (100 μM), *SB303580* (100 μM), and *IKK-NBD* control peptide (100 μM) for 1 h and then exposed to *resistin* (100 ng/mL) for 4 h. Relative luciferase activity indicates the fold expression relative to the activity of the construct co-transfected with empty vector (set as 1) in the presence of *resistin* (100 ng/mL). W/O *resistin* means blank control without *resistin*. The *p* value of *IKK-NBD* (100 μM) or *SB303580* (100 μM) was compared with *resistin* alone based on student’s *t*-test (** *p* < 0.01; *** *p* < 0.001). Each bar represents the mean ± S.D. of at least three independent experiments.

The previous results indicated that both *C/EBPβ* and *NF-κB* contributed to the transcriptional up-regulation of *CCL3* and *CCL4* [14]. To further investigate the *C/EBPβ* and *NF-κB* function on *CCL3* and *CCL4* in detail, the effect of inhibition of *C/EBPβ* and *NF-κB* was tested via inhibition of each transcription factor, separately and together. *SB303580*, an inhibitor of *C/EBPβ* via inhibition of p38MAPK [16,17], and *IKK-NBD*, a specific *NF-κB* inhibitor, reduced the expression of *CCL3* and *CCL4* with *resistin* stimulation (Figure 4A). Combining *IKK-NBD* and *SB303580* further decreased mRNA expression of *CCL3* and *CCL4* (Figure 4A).

We have indicated that the *CCL3* (−1395) and *CCL4* (−1281) constructs contain several high probability candidate *C/EBPβ* and *NF-κB* binding sites as assessed by our computational database [14]. To confirm that both transcription factors are functional, *CCL3* (−1395) and *CCL4* (−1281) promoter activities were determined after inhibition. The result showed that the promoter activities of *CCL3* and *CCL4* were significantly decreased by the inhibition of both factors, and *IKK-NBD* and *SB303580* co-enhanced the decrease of promoter activities of *CCL3* and *CCL4* (Figure 4B).

### 2.5. Immunohistochemistry of C/EBPβ and NF-κB

**Figure 5:**
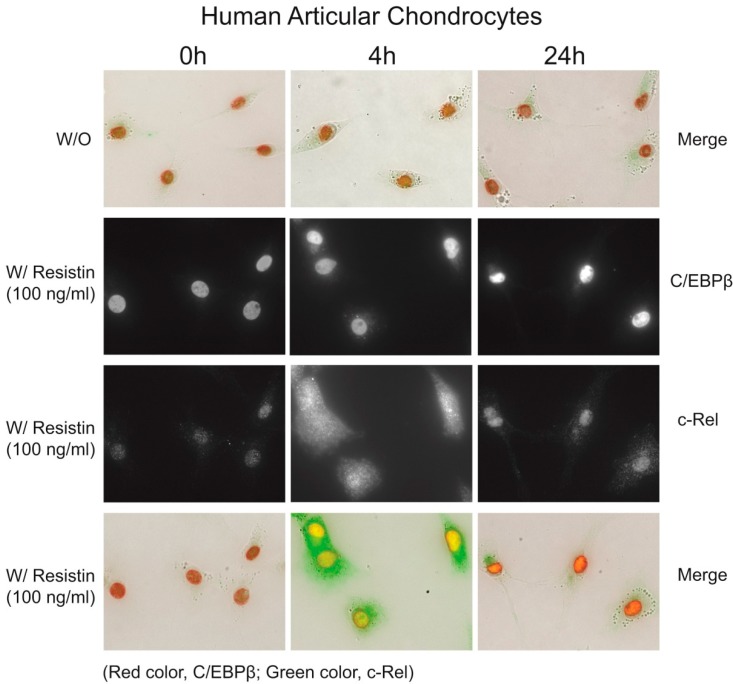
Subcellular localization of *C/EBPβ* and *c-Rel* in response to *resistin* (200×). The top panel shows a merged image of immunohistochemistry for *C/EBPβ* and the *NF-κB* subunit *c-Rel*: *C/EBPβ* is present in the nucleus of cells even without *resistin* exposure, while *c-Rel* is located diffusely throughout the cell. With addition of *resistin*, *C/EBPβ* is increased in the nucleus and *c-Rel* is increased and translocates to the nucleus. At 24 h, *C/EBPβ* remains high in the nucleus and *c-Rel* is significantly reduced.

Our biochemical studies provided evidence that the regulation of *CCL3* and *CCL4* is coordinated early in time with the *NF-κB* response, and the *C/EBPβ* response increased and sustained. To support this conclusion, we traced the location of these transcription factors over time by immunohistochemistry. We knew from Figure 3 that *p50*, *p52* and *c-Rel* exhibited the highest changes upon increasing dose of *resistin*. As we know, *p50* and *c-Rel* are involved in the canonical pathway while *p52* involved in the non-canonical pathway. However, we showed in chondrocytes that, of the *NF-κB* subunits, *c-Rel* mRNA was increased more with *resistin* stimulation, and the computational analysis in chemokine genes *CCL3* and *CCL4* showed that *c-Rel* binding sites, at −210 to −206 bp of the *CCL3* promotor (site S5) and −174 to −169 bp of the *CCL4* promotor (site S13), were predicted in the resistin-responsive elements [8]. Lu and colleagues reported that *c-Rel* was an important transcription factor in the regulation of the induction of proinflammatory cytokines [16]. Therefore, *c-Rel* would be an interesting target for further investigation in the immunohistochemistry experiment. Figure 5 demonstrated by double immunohistochemistry with antibodies to *C/EBPβ* and *c-Rel* after stimulation with *resistin*. At 0 h time, *c-Rel* was localized in the cytoplasm, then was localized in the nucleus at 4 h and was greatly reduced by 24 h. *C/EBPβ* was present at the beginning and was increased at 4 h and at 24 h.

### 2.6. Discussion

*Resistin*, a member of the adipocyte-derived cytokines, may be a potent link between adipokines and inflammatory diseases, including OA and RA [3,5,7]. Many cytokines and chemokines are up-regulated by *resistin* in human articular chondrocytes, including *IL-1β* [8]. As an important cytokine in inflammatory joint disease, *IL-1β* can: induce enzymes that degrade the extracellular matrix (ECM), reduce synthesis of the primary cartilage components *COL2A1* and *aggrecan*, and increase cytokines and chemokines [13,14]. Here, we showed that *resistin* also increased the expression of cytokines and chemokines after effective inhibition of *IL-1* receptor. Therefore, *resistin* and *IL-1β* both play a role in induction of chemokine synthesis, and *resistin* and *IL-1β* could be additive in the up-regulation of chemokine genes.

In this work, we demonstrate a crucial role of *C/EBPβ* in the increased expression of chemokines and cytokines in response to *resistin* in chondrocytes. We showed that *C/EBPβ* increased gradually in 24 h under the effect of *resistin*. We conclude from this data that *C/EBPβ* is involved in constitutive regulation of chemokine genes in response to *resistin*. From our previous work on chemokine gene stimulation and on extracellular matrix gene suppression by *C/EBPβ* [14], we hypothesize that *C/EBPβ* is one of the most important regulators of gene activity in chondrocytes in response to *resistin*. In fact, Kawaguchi and colleagues have shown that *C/EBPβ* is critical for chondrocyte hypertrophy, where it increases type X collagen and *MMP-13*, and suppresses type II collagen synthesis [18]. They also showed that the removal of *C/EBPβ* protects against osteoarthritis.

Like *C/EBPβ*, the classic pro-inflammatory mediator, *NF-κB* activity is increased by *resistin*; however, in contrast to *C/EBPβ*, the increase in *NF-κB* activity is transient. Considering the time course of expression of *CCL3* and the gradual increase of *C/EBPβ*, we suggest that *C/EBPβ* is involved in the resistin-induced up-regulation of chemokine genes over time. This is especially true for the regulation of chemokine genes like *CCL3*, which are induced more slowly, and where the *mRNA* was gradually and steadily increased, not reaching peak expression even in the 24 h observation period. In contrast, *NF-κB* appears to be involved in the resistin-induced up-regulation of chemokines in the early stage (reach the peak at 4 h), with the concentration gradually decreasing in the late stage. Co-regulation by *NF-κB* and *C/EBPβ* was recently shown in several genes in different cells [14,19,20,21]. Here, we proved *C/EBPβ* and *NF-κB* were both involved in the up-regulation of chemokine genes in response to *resistin* in human chondrocytes in a time-ordered manner: *NF-κB* provides an initial up regulation and *C/EBPβ* sustains increased gene expression.

In summary, the data presented here show that chondrocytes react in a non- restricted, cell-specific manner to *resistin*, utilizing *C/EBPβ*, *NF-κB* and some chondrogenic specific miRNAs in a combinatorial regulation of chemokine gene expression. The activity of *C/EBPβ* is augmented by a transient increase in activity of *NF-κB*, and both transcription factors act independently on the chemokine genes, *CCL3* and *CCL4*. These studies reinforce the evidence of our previous work on *resistin* and provide the foundation for the control of chemokine gene expression in chronic joint disease.

## 3. Experimental Section

### 3.1. Materials

The materials used in this work were purchased as follows: Dulbecco’s modified Eagle’s medium (DMEM), Ham’s F-12 medium, were from Mediatech Inc. (Herndon, VA, USA). Fetal bovine serum, pfx polymerase, SuperScript^®^ II Reverse Transcriptase, restriction enzymes, Alexa fluor^®^ 488 goat anti-mouse *IgG*, and Alexa fluor^®^ 594 goat anti-rabbit *IgG* from Invitrogen (Carlsbad, CA, USA); Penicillin/streptomycin solution, ascorbic acid, Actinomycin D, Tween 20 from Sigma (St. Louis, MO, USA); 16% paraformaldehyde from Electron Microscopy Science (Hatfield, PA, USA); Recombinant human *IL-1β*, and Recombinant Human *IL-1ra/IL-1F3* from R&D Systems, Inc. (Minneapolis, MN, USA); RNeasy Mini Kit, QIAshredder, DNase I, from Qiagen, Inc. (Valencia, CA, USA); FuGENE^®^ 6 Transfection Reagent, Pronase and Collagenase P from Roche (Indianapolis, IN, USA); pGL3-basic vector, Reporter Lysis Buffer, Luciferase Assay Reagent, β-galactosidase from Promega (Madison, WI, USA); anti-*C/EBPβ*, anti-*c-Rel*, normal rabbit *IgG* and Actin antibodies from Santa Cruz Biotechnology (Santa Cruz, CA, USA); SYBR Green PCR Master Mix was from Applied Biosystens (Foster City, CA, USA); Cell-permeable NEMO binding domain (*NBD*) synthetic peptides (*IKK-NBD* peptide and *IKK-NBD* control peptide), were from BIOMOL (Plymouth Meeting, PA, USA); *SB203580* was from Calbiochem (Gibbstown, NJ, USA).

### 3.2. Cell Culture

Human primary chondrocytes were obtained from articular cartilage obtained at the time of total joint replacement or from above the knee amputation, with approval of the Washington University and Sun Yat-sen University Human Studies Review Board and permission of the patient (IRB No. 05-0279, 1 July 2005 and No. 2011-029, 12 January 2011). Chondrocytes were isolated following previously published procedures [13] and plated at a density of 2.5 × 10^5^ cells/cm^2^ in DMEM/F12 media plus 10% fetal bovine serum (FBS), 50 μg/mL ascorbate and antibiotics (50 U/mL penicillin and 50 μg/mL streptomycin). *T/C-28a2* human chondrocyte cell line were also used (a gift from Dr. Mary B. Goldring, Cornell University), and cultured like human articular chondrocytes. Cells were allowed to rest for 24 h and *resistin* was added at the concentrations and times indicated. *Resistin* was reconstituted in sterile water.

### 3.3. RNA Isolation and Real Time Quantitative PCR

Total *RNA* was isolated from human primary articular chondrocytes and *T/C-28* cells with RNeasy Mini Kit with DNase I treatment, following the protocol recommended by the manufacturer (Qiagen). Total *RNA* (1 μg) was reverse-transcribed with a SuperScriptTM II Reverse Transcriptase to synthesize *cDNA*. The *cDNA* was then used for real-time quantitative PCR (qPCR). Real-time quantitative PCR was performed in a total volume of 20 μL reaction mixture containing 10 μL of SYBR Green PCR Master Mix, 2.5 μL of cDNA, and 200 nM of primers using a 7300 Real-Time PCR System (Applied Biosystems, Foster City, CA, USA), and done in triplicate. Primers used for qPCR were optimized for each gene, and the dissociation curve was determined by the Real-Time PCR System. The parameters of primer design included a primer size of 18 to 21 bp, a product size of 80 to 150 bp, a primer annealing temperature of 59° to 61°, and a primer GC content of 45% to 55%. Results were normalized to glyceraldehyde-3-phosphate dehydrogenase (*GAPDH*). The primer sequences are summarized in Table 1. The cycle threshold (*C*_t_) values for *GAPDH* and those of genes of interest was measured for each sample, and the relative transcript levels were calculated as χ = 2^−ΔΔ*C*t^, in which ∆∆*C*_t_ = ∆Treatment − ∆*C* and ∆Treatment = *C*_t_(treatment) − *C*_t_(*GAPDH*); ∆*C* = *C*_t_(control) − *C*_t_(*GAPDH*).

**Table 1:** Primers for real-time quantitative PCR.

Gene	Primer Sequence
*GAPDH*	F(5'-ACCCAGAAGACTGTGGATGG-3'), R(5'-GAGGCAGGGATGATGTTCTG-3')
*COL2A*1	F(5'-CCCAGAGGTGACAAAGGAGA-3'), R(5'-CACCTTGGTCTCCAGAAGGA-3')
*Aggrecan*	F(5'-GGCACTAGTCAACCCTTTGG-3'), R(5'-CTGAACCCTGGTAACCCTGA-3')
*C/EBPβ*	F(5'-CTCGCAGGTCAAGAGCAAGG-3'), R(5'-TCGTCGCTGTGCTTGTCC-3')
*CCL3 (MIP-1α)*	F(5'-GCAACCAGTTCTCTGCATCA-3'), R(5'-TGGCTGCTCGTCTCAAAGTA-3')
*CCL3L1 (LD78β)*	F(5'-GTCCTCTCTGCACCACTTGC-3'), R(5'-GGAAGATGACACTGGGCTTG-3')
*CCL4 (MIP-1β)*	F(5'-GCTTTTCTTACACTGCGAGGA-3'), R(5'-CCAGGATTCACTGGGATCAG-3')
*CCL20 (MIP-3α)*	F(5'-GCTTTTCTTACACTGCGAGGA-3'), R(5'-CCAGGATTCACTGGGATCAG-3')
*CXCL1 (GRO-α)*	F(5'-GGGAATTCACCCCAAGAAC-3'), R(5'-GATGCAGGATTGAGGCAAG-3')
*CXCL2 (GRO-β)*	F(5'-TGCAGGGAATTCACCTCAAG-3'), R(5'-TCTTAACCATGGGCGATGC-3')
*CXCL3 (GRO-γ)*	F(5'-ACCGAAGTCATAGCCACACTC-3'), R(5'-GGTGCTCCCCTTGTTCAGTA-3')
*CXCL6 (GCP-2)*	F(5'-GTTTACGCGTTACGCTGAGAG-3'), R(5'-ACTTCCACCTTGGAGCACTG-3')
*IL-8*	F(5'-GAAGGTGCAGTTTTGCCAAG-3'), R(5'-TGTGGTCCACTCTCAATCACTC-3')
*IL-1α*	F(5'-TGCCTGAGATACCCAAAACC-3'), R(5'-AACAAGTTTGGATGGGCAAC-3')
*IL-1β*	F(5'-TCCAGGAGAATGACCTGAGC-3'), R(5'-GTGATCGTACAGGTGCATCG-3')
*NF-κB1 (p50)*	F(5'-CCTGGATGACTCTTGGGAAA-3'), R(5'-TCAGCCAGCTGTTTCATGTC-3')
*NF-κB2 (p52)*	F(5'-GAACAGCCTTGCATCTAGCC-3'), R(5'-TTTTCAGCAT GGATGTCAGC-3')
*p65 (RelA)*	F(5'-TCTGCTTCCAGGTGACAGTG-3'), R(5'-GCCAGAGTTTCGGTTCACTC-3')
*c-Rel*	F(5'-CGAACCCAATTTATGACAAC-3'), R(5'-TTTTGTTTCTTTGCTTTATTGC-3')
*RelB*	F(5'-CTGCTTCCAGGCCTCATATC-3'), R(5'-CGCAGCTCTGATGTGTTTGT-3')
*IκBα*	F(5'-GATCCGCCAGGTGAAGGG-3'), R(5'-GCAATTTCTGGCTGGTTGG-3')

### 3.4. Plasmid Constructs

A series of *CCL3* and *CCL4* promoter 5'-deletion constructs were made by PCR and subcloned into pGL3-basic vector using the *pGL2-CCL3* (−1972/+75) and *pGL3-CCL4* (−1281/+12) as previously described [22,23]. The *CCL3* and *CCL4* promoter constructs, *C/EBPβ* and IkappaB kinase 2 (*IKK2*) expression vector, and *pNF-κB* luciferase reporter were provided by the following: the human *pGL2-CCL3* (−1972/+75) was from Dr. G. David Roodman (University of Pittsburgh) [15]; human *pGL3-CCL4* (−1281/+12) was obtained from Dr. Sheau-Farn Yeh (National Yang-Ming University, Taipei, Taiwan) [17]; human *IKK2* in the *pCDNA3* vector and *pNF-κB* luciferase reporter were from Dr. Yousef Abu-Amer (Washington University) [24]; human C/EBP-full-length in the *pCDNA3* vector was from Dr. Erika Crouch (Washington University, Washinton, DC, USA) [25]. The empty expression vectors were made by excision of *cDNAs* from the corresponding *C/EBP* expression vectors [15]. To facilitate subcloning of the amplified fragments, the antisense primer contained a *Hin*dIII restriction site adaptor, and the sense primer contained an XhoI or SmaI site. The PCR fragments and the luciferase expression vector pGL3-basic vector were digested with *Xho*I or *Sma*I and *Hin*dIII before ligation. All constructs were confirmed by DNA sequence analysis using a GL2 primer and RV3 primer.

### 3.5. Transient Transfection and Luciferase Assay

DNA transfections of *T/C-28a2* cells were performed using *FuGENE* 6TM transfection reagent. One hundred and five × two of *T/C-28a2* cells were cultured in a 6-well plate overnight. The transfection mixture containing 3–9 μL of transfection reagent (6:1 ratio of trasfection reagent μL to DNA μg), 500 ng of various promoter constructs, and 100 or 200 ng of pCMV-β-gal was then added, and the cells were cultured for 4 h with or without *resistin* as indicated. For co-transfection assay, 500 ng of *C/EBPβ* expression vectors or empty vector were added to the 100 μL transfection mixture as indicated. Due to low translation efficiency of *C/EBPβ*, we used higher amounts of plasmid (500 ng) for transfection, as shown previously [9]. FBS was added to transfection medium 4 h later to a final concentration of 10%. After 24 h of incubation, cells were replaced with fresh complete medium and incubated for an additional 4 h with or without *resistin*. The cells were then harvested with Reporter Lysis Buffer™ and the lysate was analyzed for luciferase activity using Promega Luciferase Assay Reagent™. The β-galactosidase activities were also measured to normalize variations in transfection efficiency. Each transfection experiment was performed in duplicate or triplicate and repeated at least three times.

### 3.6. Immunofluorescence

Five × one hundred and four human articular chondrocytes were cultured in each well of 8-well chamber slides (from Lab Tek, Hatfield, PA, USA). Cells were allowed to rest for 24 h and *resistin* was then added at the times indicated. Cells were incubated in 4% paraformaldehyde in PBS for 10 min, 0.2% Triton-X-100 in PBS for 5 min, and 10% normal goat serum in PBS for 2 h at room temperature. Cells were reacted with rabbit anti-*C/EBPβ* and mouse anti-*c-Rel* antibodies diluted to 1:400 in 2% normal goat serum in PBS for overnight at 4 °C. The secondary antibodies, Alexa fluor 488 dye-labeled goat anti-mouse IgG diluted to 1:250 and Alexa fluor 594 dye-labeled goat anti-rabbit IgG diluted to 1:400 in 2% normal goat serum in PBS were then added to the cells for 1 h at room temperature. Immunoreactivity was detected by fluorescence microscopy.

### 3.7. Statistical Analysis

Data were expressed as the mean ± S.E.M. from at least three independent experiments. The Student’s *t*-test was used to compare the differences between two groups. *p* < 0.05 was considered statistically significant.

## 4. Conclusions

Chondrocytes react in a non-restrictedly cell-specific manner to *resistin*, utilizing *C/EBPβ* and *NF-κB* in a combinatorial regulation of chemokine gene expression. The activity of *C/EBPβ* is augmented by a transient increase in activity of *NF-κB*, and both transcription factors act independently on the chemokine genes, *CCL3* and *CCL4*.
